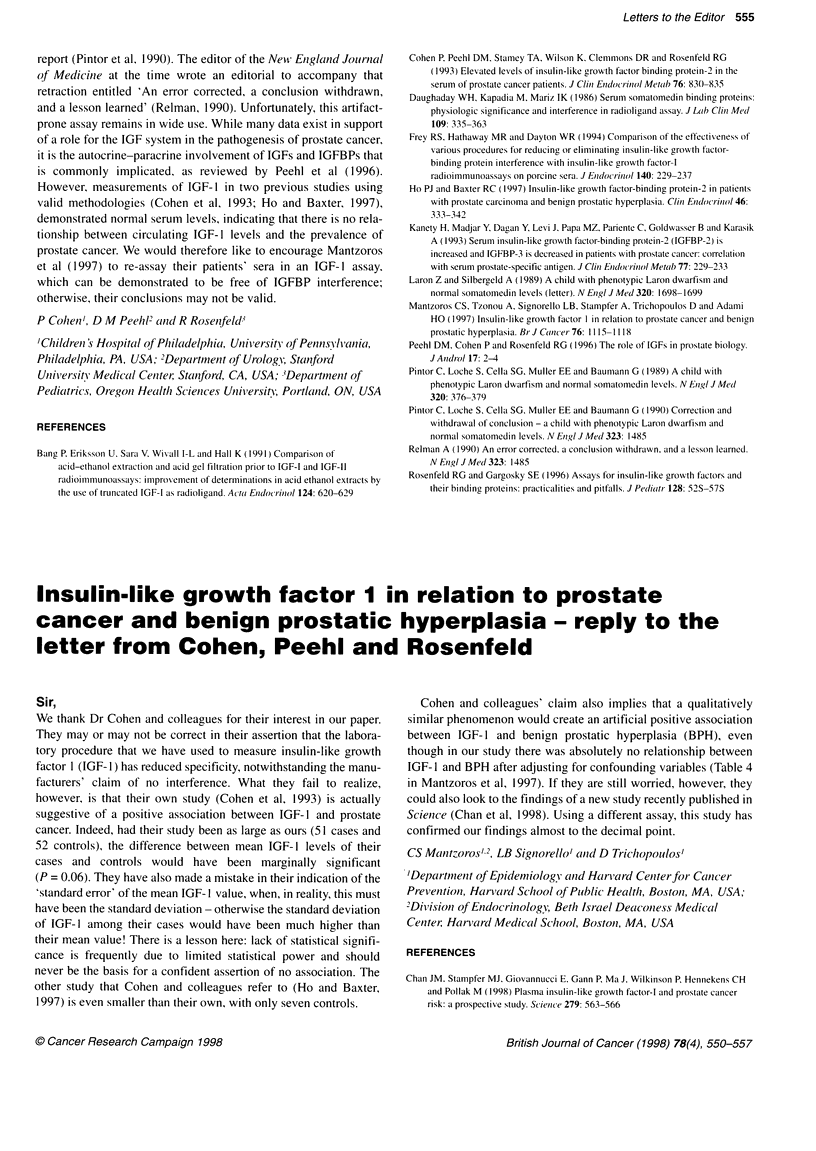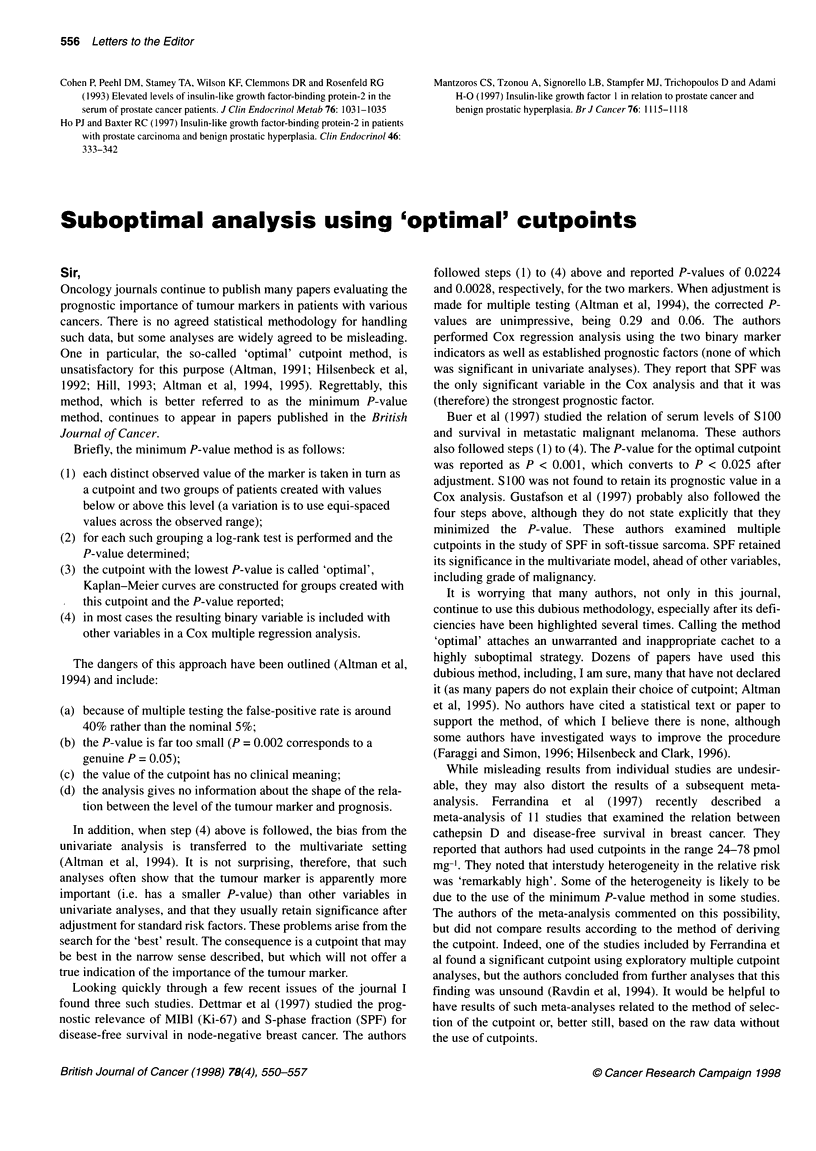# Insulin-like growth factor 1 in relation to prostate cancer and benign prostatic hyperplasia - reply to the letter from Cohen, Peehl and Rosenfeld

**Published:** 1998-08

**Authors:** CS Mantzoros, LB Signorello, D Trichopoulos


Ho PJ and Baxter RC (1997) Insulin-like growth factor-binding protein-2 in patients with prostate carcinoma and benign prostatic hyperplasia. *Clin Endocrinol* **46**: 333–342

### Insulin-like growth factor 1 in relation to prostate cancer and benign prostatic hyperplasia – reply to the letter from Cohen, Peehl and Rosenfeld

**Sir,**

We thank Dr Cohen and colleagues for their interest in our paper. They may or may not be correct in their assertion that the laboratory procedure that we have used to measure insulin-like growth factor 1 (IGF-1) has reduced specificity, notwithstanding the manufacturers' claim of no interference. What they fail to realize, however, is that their own study (Cohen et al, 1993) is actually suggestive of a positive association between IGF-1 and prostate cancer. Indeed, had their study been as large as ours (51 cases and 52 controls), the difference between mean IGF-1 levels of their cases and controls would have been marginally significant ($P = 0.06$). They have also made a mistake in their indication of the 'standard error' of the mean IGF-1 value, when, in reality, this must have been the standard deviation – otherwise the standard deviation of IGF-1 among their cases would have been much higher than their mean value! There is a lesson here: lack of statistical significance is frequently due to limited statistical power and should never be the basis for a confident assertion of no association. The other study that Cohen and colleagues refer to (Ho and Baxter, 1997) is even smaller than their own, with only seven controls.

Cohen and colleagues' claim also implies that a qualitatively similar phenomenon would create an artificial positive association between IGF-1 and benign prostatic hyperplasia (BPH), even though in our study there was absolutely no relationship between IGF-1 and BPH after adjusting for confounding variables (Table 4 in Mantzoros et al, 1997). If they are still worried, however, they could also look to the findings of a new study recently published in *Science* (Chan et al, 1998). Using a different assay, this study has confirmed our findings almost to the decimal point.

*CS Mantzoros[1,2], LB Signorello[1] and D Trichopoulos[1]*

*[1]Department of Epidemiology and Harvard Center for Cancer Prevention, Harvard School of Public Health, Boston, MA, USA; [2]Division of Endocrinology, Beth Israel Deaconess Medical Center, Harvard Medical School, Boston, MA, USA*

### REFERENCES

Chan JM, Stampfer MJ, Giovannucci E, Gann P, Ma J, Wilkinson P, Hennekens CH and Pollak M (1998) Plasma insulin-like growth factor-I and prostate cancer risk: a prospective study. *Science* **279**: 563–566

© Cancer Research Campaign 1998

Cohen P, Peehl DM, Stamey TA, Wilson KF, Clemmons DR and Rosenfeld RG (1993) Elevated levels of insulin-like growth factor-binding protein-2 in the serum of prostate cancer patients. *J Clin Endocrinol Metab* **76**: 1031–1035
Ho PJ and Baxter RC (1997) Insulin-like growth factor-binding protein-2 in patients with prostate carcinoma and benign prostatic hyperplasia. *Clin Endocrinol* **46**: 333–342
Mantzoros CS, Tzonou A, Signorello LB, Stampfer MJ, Trichopoulos D and Adami H-O (1997) Insulin-like growth factor 1 in relation to prostate cancer and benign prostatic hyperplasia. *Br J Cancer* **76**: 1115–1118